# Occurrence of Tungiasis in Ceará State, Northeast Brazil: Results of a Rapid Assessment Method

**DOI:** 10.1590/0037-8682-0274-2023

**Published:** 2023-11-10

**Authors:** Nathiel de Sousa Silva, Carlos Henrique Alencar, Cláudia Maria Lins Calheiros, Jorg Heukelbach

**Affiliations:** 1 Universidade Federal do Ceará, Faculdade de Medicina, Programa de Pós-graduação em Saúde Pública, Fortaleza, CE, Brasil.; 2 Universidade Federal de Alagoas, Instituto de Ciências Biológicas e da Saúde, Maceió, AL, Brasil.

**Keywords:** Tungiasis, Ceará, Brazil, Rapid Assessment, Epidemiology, Neglected Tropical Disease

## Abstract

**Background:**

Systematic data on the occurrence of tungiasis are scarce.

**Methods:**

We analyzed data on tungiasis in humans and animals from all municipalities of Ceará State, Northeast Brazil, using a rapid assessment questionnaire.

**Results:**

Among the 184 municipalities, 181 (98.3%) reported tungiasis in the past (before 2021) or currently, 120 (65.2%) reported current occurrence, 155 (84.2%) reported severe cases in the past or currently, 47 (25.5 %) reported severe cases currently, and 132 (71.7%) reported tungiasis in animals.

**Conclusions:**

Tungiasis is a significant public health concern in Ceará. A One Health approach is required to reduce the disease burden in humans and animals.

Despite the recent recognition of tungiasis as a Neglected Tropical Disease by the World Health Organization (WHO) in 2017 and its widespread occurrence in Latin America, Sub-Saharan Africa, and the Caribbean, systematic data on the occurrence and severity of this disease are still scarce^1^. A recent study based on ecological niche modeling estimated that, in 17 Latin American countries, approximately 450 million people are living in areas at risk for tungiasis; however, this study used published data spanning several decades^2^. Some population-based studies reported a prevalence of 23-54% in humans, and reports exist on secondary cases of tetanus and even deaths from tungiasis, mainly in small children and older adults^1^. High prevalence and severe tungiasis are usually related to poor living conditions, lack of disease control measures in domestic and synanthropic animals, presence of reservoir animals close to humans, and individual behavior (such as no regular use of footwear and use of common resting places)^1^ ^,^ ^3^. Commonly described risk factors for tungiasis also include other socioeconomic variables, younger and older age, and male sex; however, their interactions and importance vary based on setting and are a matter of debate^4^. 

A rapid assessment method to assess the prevalence based on visual inspection of the periungual region of the feet has been developed and validated in Brazil and Nigeria^5^, which requires field visits and quick inspection of individual feet. This may be a major challenge in settings with limited human and financial resources and wide geographic range. Obtaining information from key informants, schoolteachers, and healthcare networks may thus be a more cost-effective and quicker for estimating general disease occurrence and severity at the municipality level. 

In 2021, an informal expert meeting, headed by the WHO Department of Control of Neglected Tropical Diseases and the PAHO Regional Program of Neglected Infectious Diseases, was held to develop a conceptual framework for tungiasis. Various aspects of tungiasis and sustainable control were discussed and a consensus with recommendations was reached. Experts in the meeting raised awareness that the information status on disease mapping, surveillance, prevention and treatment is “*desperate*”^6^. 

To fill this gap, we developed and applied a rapid assessment method in Ceará State in Northeast Brazil, based on an open online questionnaire for healthcare professionals and other key stakeholders and that can be used through mobile phones or other mobile devices to obtain basic data on tungiasis^7^ ^.^ Here, we present an analysis of this statewide assessment, as a basis for planning evidence-based control measures and in-depth field studies.

Ceará State consists of 184 municipalities and has an area of 149,000 km^2^ (population: approximately 9.2 million) (Figure 1). The climate is predominantly semiarid, with irregular rainfall, usually concentrated over 3 months (February to April). Previous population-based studies have shown the occurrence of tungiasis in several municipalities of the state^5^ ^,^ ^8^; however, the disease distribution throughout these municipalities is unknown. 

**FIGURE 1:**
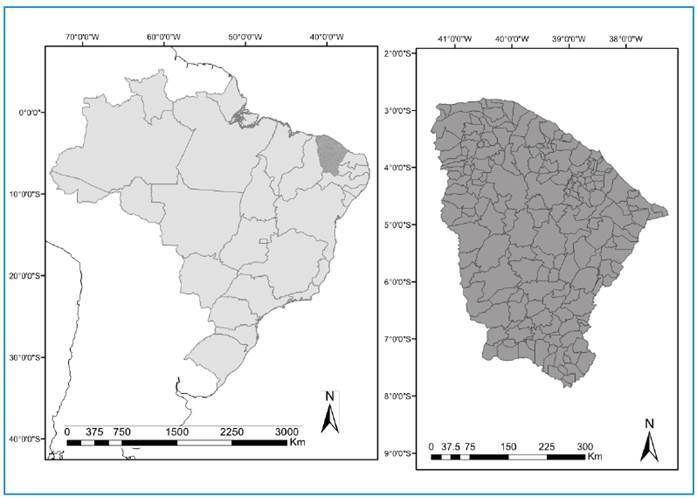
Location of Ceará State in Brazil (left) and distribution of the 184 municipalities (right).

Data were collected using an online rapid assessment survey tool from March to September 2021, as described in detail previously^7^. The Federation of Community Health Workers of Ceará and Syndicate of Health Workers of Ceará collaborated to redirect the questionnaire link to health professionals, thus initiating snowball sampling. We also successfully contacted the municipal coordinators of health teams and units in the two silent municipalities that redirected the questionnaire link to their health staff. 

The questions were on the occurrence of tungiasis and severe tungiasis, its seasonality, and presence of specific disease control programs (questionnaire available at www.bit.ly/ectoparasitoses). The main respondents were Community Health Agents. Other health professionals such as nurses, physicians, nurse technicians, veterinarians, and endemic disease agents also responded. We did not perform a clinical study but aimed to describe the occurrence and morbidity of tungiasis as subjectively perceived and observed by key informants. 

Overall, 1,265 valid individual data entries were available, with each municipality having 1-104 entries. The present analysis grouped individual answers per municipality and considered the municipalities as observation units. Individual entries were merged into a single dataset per municipality. This study was approved by the Ethical Review Board of the Federal University of Ceará.

Overall, 181/184 (98.3%) municipalities reported the occurrence of tungiasis in the past (before 2021) or at present and 120 (65.2%) reported current occurrence (Table 1). Three municipalities (Iracema, Larvras da Mangabeira and Várzea Alegre) did not report any tungiasis cases. However, only 1-3 responses (online data entries) were available for these municipalities. Overall, 155 (84.2%) municipalities reported severe tungiasis cases in the past (before 2021) or currently, and 47 (25.5%) reported severe cases.

**TABLE 1:** Municipalities reporting the occurrence of tungiasis and severe tungiasis in humans and of tungiasis in animals.

	Number of municipalities (n=184)	%
Occurrence of tungiasis		
Currently	120	65.2
In the past or currently	181	98.3
Occurrence of severe tungiasis		
Currently	47	25.5
In the past or currently	155	84.2
Tungiasis in animals	132	71.7
Severe tungiasis in animals	115	62.5
Observation of seasonality	146	79.3
Specific control programs	0	-

Respondents subjectively described the severe cases observed in their communities as: *“amputation of fingers and toes,” “simultaneous lesions in an entire family,” “multiple injuries on a child’s hands,” “secondary infection,” “person with walking difficulties,” “amputation of the toes of a lady with diabetes,” “lesions on the buttocks,” “loss of nails,” “children who needed hospital care to remove fleas,” “death due to disease worsening,” “hospitalization,” and “children from pig-raising families who did not have adequate family support and who needed the guardianship council to refer the case to hospital care.”*

Approximately 72% of the municipalities reported tungiasis in animals (Table 1). The affected animal species were mainly dogs (n=97) and pigs (n = 80), followed by cats (n = 50), horses (n = 20), cattle (n = 8), and goats (n = 6). In animals, severe cases were described based on personal observations as: *“tungiasis leading to death,” “swollen and bleeding paws,” “pigs with exaggerated lesions,” “occurrence of secondary infections,” “mutilation,” “loss of ability to walk,” “loss of nails,” “apathy,” “loss of weight and appetite,” “lesions on teats,” “loss of the hoof,” “loss of limbs.”*

Most respondents from the municipalities reported seasonality of tungiasis. No report exists on any specific disease control program in the state of Ceará. Figure 2 depicts the spatial distribution of municipalities regarding the current and past occurrences of tungiasis, severe tungiasis, and its occurrence in animals.

**FIGURE 2:**
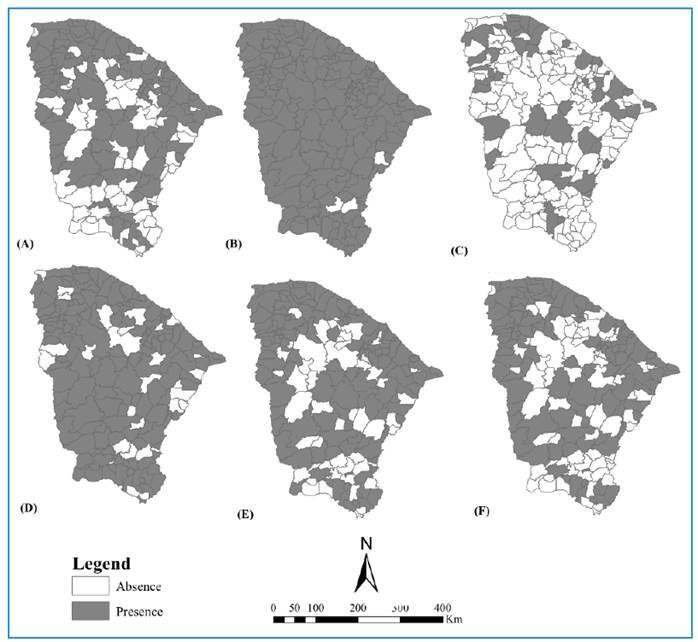
Spatial distribution of municipalities reporting **(A)** tungiasis in the present and **(B)** in the present or past, **(C)** severe disease in the present and **(D)** in the present or past, **(E)** tungiasis in animals, and **(F)** severe disease in animals.

This is the first systematic study on the occurrence of tungiasis, covering a broad and complete geographic area. We describe tungiasis patterns in Ceará based on primary data as well as the severity and temporal, zoonotic, and seasonal aspects. Although the results are based on subjective information from key informants and the correctness of clinical diagnoses is unverified, they nonetheless indicate the common occurrence of tungiasis and severe tungiasis in Ceará state, highlighting it as a significant public health issue in the region. Tungiasis and severe tungiasis occurred in all regions of Ceará State, independent of the predominant ecosystem for both humans and animals. 

Health professionals from many municipalities described severe cases, indicating an urgent need for the implementation of systematic control measures. In cases of massive infection, chronic inflammation, difficulty in walking, deformation of digits, loss of toenails, and superinfection with pathogenic bacteria are common^4^ ^,^ ^9^. During the past decades, mostly because of increasing urbanization and better living conditions, the number of affected municipalities and those reporting severe cases have decreased. 

Tungiasis of zoonotic origin is an important transmission factor in endemic communities. In our study, domestic animals such as dogs, pigs, and cats were most commonly mentioned as animal reservoirs for *T. penetrans*; in fact these are commonly considered important animal reservoirs^1^ ^,^ ^4^ ^,^ ^10^. In Brazil, ownership of infected semi-restricted dogs and cats is associated with a higher intensity of infection and number of infected individuals in the household; infection rates in humans and domestic animals are also correlated within the same household^1^. 

However, farm animals have also been affected, posing an economic threat to owners. Severe diseases in farm animals, possibly leading to considerable economic loss, were described by the respondents, including loss of limbs and infections, leading to death. Rats and sylvatic animals may also serve as reservoirs. Additionally, in the rural areas of Ceará, keeping sylvatic animals, such as common marmosets, as pets is common practice. 

Under favorable temperature and humidity conditions, eggs expelled from female fleas develop into larvae and pupae. The fleas then infect humans and domestic and sylvatic animals, further spreading the ectoparasite in communities. Consequently, the presence of a vast variety of animal host species of *T. penetrans* is a major challenge for sustainable control, particularly considering the interaction between domestic, synanthropic and wild animals in both rural and urban settings. Control strategies would thus require not only control of off-host stages (i.e., eggs, larvae, and pupae) in the environment but also disease control in the animal host species, in addition to controlling infections in affected humans^1^. In resource-poor communities, high attack rates are observed on compounds and inside houses as a consequence of precarious housing conditions, presence of animals, abundant waste scattered around, and low educational levels. In addition to the focus on human and animal reservoirs, off-host stages need to be reduced, and high-density breeding sites need to be eliminated in communities and inside houses. This will considerably increase the quality of life of the affected populations. Thus, a variety of disciplines within the One Health approach should work together^11^. These would include not only human and veterinary medicine but also biology, architecture, and social sciences, among others^4^. 

The seasonality of tungiasis occurrence is well reported^9^ ^,^ ^12^ ^-^ ^13^. Usually, fewer cases occur during the rainy season; higher incidences, and consequently, higher prevalence and morbidities occur during the dry season. Indeed, many respondents in our study were aware of this phenomenon, and respondents reported an increase in the number of cases during the dry season. Control measures should be planned and scheduled in line with this seasonal variation to reduce the number of severe cases during the high transmission season.

There are several examples of the successful and sustainable control of tungiasis. For example, in a fishing community in Brazil, treatment of affected individuals and spraying of insecticide in the premises resulted in reduced prevalence, although short-lived^8^. A more recent report from a rural community in Nigeria was based on a low-cost interdisciplinary community-based One-Health approach that resulted in sustainable control^3^. In Kenya, a local non-governmental organization (NGO) managed to control tungiasis through intensive cooperation with communities and policy makers^14^. Policymakers, communities, and healthcare professionals should work together to create sustainable tungiasis control programs. However, integrated multiprofessional activities may be hindered by continuous silo thinking, and the implementation of the One Health concept may pose major challenges in political practice^4^ ^,^ ^15^. 

Our study has some limitations. First, this study was intended to provide basic information on the occurrence of disease and severe disease in a large geographic area, and thus does not substitute population-based field studies. The clinical diagnosis of severe cases was not the objective of this study. Second, there was a considerable variety of respondents by municipality, and the municipality sizes differed considerably. The number of responses may have been lower in areas with low endemicity. It can be assumed that tungiasis may also occur (or have occurred in the past) in some of the municipalities with few responses, as these share similar socioeconomic, climatic, and ecological characteristics compared with neighboring municipalities that reported tungiasis. Many community health agents were heavily involved in measures directed towards controlling the coronavirus disease (COVID-19) pandemic, which may have reduced their participation in several municipalities. 

In conclusion, based on a new rapid assessment method with key informants as the data source, our study estimated the geographic distribution of tungiasis and severe tungiasis in both humans and animals in Ceará State for the first time. These results fill the gap in the missing data on the geographic distribution of tungiasis in Northeast Brazil. No single municipality had an established disease control program, highlighting the need for further action and policy changes. In-depth studies should be performed, focusing on the most affected areas within the municipalities, to target them for intervention measures and to verify and describe in detail the occurrence of severe diseases in high-risk groups. Given the complex life cycle of the parasite and abundance of animal reservoir species, a multisectoral One Health approach is of paramount importance. Not only humans and domestic animals but also the environment, wildlife, housing conditions, and social sciences should be considered when planning intervention measures. 

## References

[1] Harvey T, Linardi PM, Carlos RSA, Heukelbach J (2021). Tungiasis in domestic, wild, and synanthropic animals in Brazil. Acta Tropica.

[2] Deka MA, Heukelbach J (2022). Distribution of Tungiasis in Latin America: Identification of Areas for Potential Disease Transmission Using an Ecological Niche Model. Lancet Reg Health Am.

[3] Heukelbach J, Ariza L, Adegbola RQ, Ugbomoiko US (2021). Sustainable control of tungiasis in rural Nigeria: a case for One Health. One Health Implement Res.

[4] Heukelbach J, Harvey TV, Calheiros CML, Mehlhorn H, Heukelbach J (2022). Infectious Tropical Diseases and One Health in Latin America. Parasitology Research Monographs.

[5] Ariza L, Wilcke T, Jackson A, Gomide M, Ugbomoiko US, Feldmeier H (2010). A simple method for rapid community assessment of tungiasis. Trop Med Int Health.

[6] World Health Organization (WHO) (2022). Report of a WHO informal meeting on the development of a conceptual framework for tungiasis control: virtual meeting, 11-13, January 2021.

[7] Heukelbach J, Silva NS (2023). A rapid assessment method for the estimation of the occurrence of epidermal parasitic skin diseases in Brazil: tungiasis and scabies as case studies. One Health Implement Res.

[8] Pilger D, Schwalfenberg S, Heukelbach J, Witt L, Mencke N, Khakban A (2008). Controlling tungiasis in an impoverished community: An intervention study. Plos Negl Trop Dis.

[9] Saboyá-Díaz MI, Nicholls RS, Castellanos LG, Feldmeier H (2022). Current status of the knowledge on the epidemiology of tungiasis in the Americas. Rev Panam Salud Publica.

[10] Ugbomoiko US, Ariza L, Heukelbach J (2008). Pigs are the most important animal reservoir for Tunga penetrans (jigger flea) in rural Nigeria. Trop Doct.

[11] Heukelbach J (2020). One health & implementation research: improving health for all. One Health Implement Res.

[12] Heukelbach J, Wilcke T, Harms SG, Feldmeier H (2005). Seasonal variation of tungiasis in an endemic community. Am J Trop Med Hyg.

[13] Heukelbach J, Jackson A, Ariza L, Calheiros C, Soares V, Feldmeier H (2007). Epidemiology and clinical aspects of tungiasis (sand flea infestation) in Alagoas State, Brazil. J Infect Dev Ctries.

[14] Elson L, Wright K, Swift J, Feldmeier H (2017). Control of tungiasis in absence of a roadmap: grassroots and global approaches. Trop Med Infect Dis.

[15] Gaviria A (2021). My experience with One Health: between realism and optimism. One Health Implement Res.

